# Reporting of costs and economic impacts in randomized trials of de-implementation interventions for low-value care: a systematic scoping review

**DOI:** 10.1186/s13012-023-01290-3

**Published:** 2023-08-21

**Authors:** Petra Falkenbach, Aleksi J. Raudasoja, Robin W. M. Vernooij, Jussi M. J. Mustonen, Arnav Agarwal, Yoshitaka Aoki, Marco H. Blanker, Rufus Cartwright, Herney A. Garcia-Perdomo, Tuomas P. Kilpeläinen, Olli Lainiala, Tiina Lamberg, Olli P. O. Nevalainen, Eero Raittio, Patrick O. Richard, Philippe D. Violette, Kari A. O. Tikkinen, Raija Sipilä, Miia Turpeinen, Jorma Komulainen

**Affiliations:** 1 Finnish Coordinating Center for Health Technology Assessment, Oulu University Hospital, University of Oulu, Oulu, Finland; 2 Finnish Medical Society Duodecim, Faculty of Medicine, University of Helsinki, Helsinki, Finland; 3 Department of Nephrology and Hypertension, University Medical Center Utrecht, Utrecht, The Netherlands; 4 Julius Center for Health Sciences and Primary Care, University Medical Center Utrecht, Utrecht University, Utrecht, The Netherlands; 5 Mehiläinen Oy, Helsinki, Finland; 6 Department of Medicine, Division of General Internal Medicine, McMaster University, Hamilton, ON Canada; 7 Department of Health Research Methods, Evidence and Impact, McMaster University, Hamilton, ON Canada; 8 Department of Urology, University of Fukui Faculty of Medical Sciences, Fukui, Japan; 9 Department of General Practice and Elderly Care Medicine, University Medical Centre Groningen, University of Groningen, Groningen, The Netherlands; 10 Department of Gynaecology, Chelsea and Westminster NHS Foundation Trust, London, UK; 11 Department of Surgery, Division of Urology/Uro-Oncology, School of Medicine, Universidad del Valle, Cali, Colombia; 12 Department of Urology, University of Helsinki and Helsinki University Hospital, Helsinki, Finland; 13 Department of Radiology, Faculty of Medicine and Health Technologies, Imaging Centre, Tampere University Hospital, Tampere University, Tampere, Finland; 14 Finnish Medical Society Duodecim, Helsinki, Finland; 15 Wellbeing Services County of Pirkanmaa, Unit of Health Sciences, Faculty of Social Sciences, Hatanpää Health Center, Tampere University, Tampere, Finland; 16 Department of Dentistry and Oral Health, Oral Health Care, Institute of Dentistry, Aarhus University, University of Eastern, Kuopio, Finland; 17 Division of Urology, Centre Hospitalier Universitaire de Sherbrooke, Sherbrooke, Canada; 18 Departments of Surgery and Health Research Methods Evidence and Impact, McMaster University, Hamilton, Canada; 19 Department of Surgery, South Karelian Central Hospital, Lappeenranta, Finland; 20 Oulu University Hospital, University of Oulu, Oulu, Finland

**Keywords:** De-implementation, Systematic scoping review, Cost, Health care costs, De-implementation strategy

## Abstract

**Background:**

De-implementation of low-value care can increase health care sustainability. We evaluated the reporting of direct costs of de-implementation and subsequent change (increase or decrease) in health care costs in randomized trials of de-implementation research.

**Methods:**

We searched MEDLINE and Scopus databases without any language restrictions up to May 2021. We conducted study screening and data extraction independently and in duplicate. We extracted information related to study characteristics, types and characteristics of interventions, de-implementation costs, and impacts on health care costs. We assessed risk of bias using a modified Cochrane risk-of-bias tool.

**Results:**

We screened 10,733 articles, with 227 studies meeting the inclusion criteria, of which 50 included information on direct cost of de-implementation or impact of de-implementation on health care costs. Studies were mostly conducted in North America (36%) or Europe (32%) and in the primary care context (70%). The most common practice of interest was reduction in the use of antibiotics or other medications (74%). Most studies used education strategies (meetings, materials) (64%). Studies used either a single strategy (52%) or were multifaceted (48%). Of the 227 eligible studies, 18 (8%) reported on direct costs of the used de-implementation strategy; of which, 13 reported total costs, and 12 reported per unit costs (7 reported both). The costs of de-implementation strategies varied considerably. Of the 227 eligible studies, 43 (19%) reported on impact of de-implementation on health care costs. Health care costs decreased in 27 studies (63%), increased in 2 (5%), and were unchanged in 14 (33%).

**Conclusion:**

De-implementation randomized controlled trials typically did not report direct costs of the de-implementation strategies (92%) or the impacts of de-implementation on health care costs (81%). Lack of cost information may limit the value of de-implementation trials to decision-makers.

**Trial registration:**

OSF (Open Science Framework): https://osf.io/ueq32.

**Supplementary Information:**

The online version contains supplementary material available at 10.1186/s13012-023-01290-3.

Contributions to the literature

- The need for economic information has been identified in the field of implementation science. The same should be expected for studies reporting de-implementation strategies.
- The costs and impact on health care costs of de-implementation strategies are currently seldom reported in randomized trials on de-implementation. Even when they are reported, the information is incomplete and scarce.
- To improve health care quality and effective resource use, de-implementation strategies need to measure clinically relevant outcomes, and the trials also need to report intervention costs and impact on health care costs.

## Background

Efficient use of health resources benefits both individuals and society — and one way to increase efficiency is to abandon obsolete and ineffective health interventions [1, 2]. De-implementation is typically aimed at reducing the use of low-value care, which has been described as providing little or no benefit, being potentially harmful, and leading to unnecessary costs to patients or wasting health care resources [3]. To achieve this, de-implementation strategies are needed. De-implementation, a process to reduce the use of a medical practice, can occur in four different ways, by removing, replacing, reducing, or restricting the use [4]. Each category has different underlying reasons, and therefore, different solutions may be needed [5]. It is easier to implement new interventions than it is to de-implement existing medical practices [6].

Different terms are used from these withdrawn actions, like de-implementation and disinvestment [7]. Public policy concepts, like disinvestment, are relevant to de-implementation study. Many de-implementation frameworks and models mention costs as a justification for de-implementation [8]. De-implementation has the potential to decrease health care costs [5, 7, 9], and bringing these out requires an evaluation of clinical practices and care pathways. De-implementation can increase health care costs but is still cost cutting to the society. Health technology assessment is one way to assess these changes in clinical practices and care pathways [10]. Differences between health care systems in different countries affect clinical practices and care pathways and costs, which must be taken into account when transferring information from one country to another.

Economic evaluation has been pointed out to be crucial part of implementation research [11–16], and costs are identified as a key outcome in implementation research [13, 17]. An economic evaluation can bring out whether using a strategy to improve the quality of health care is a cost-effective use of limited resource [13]. Without knowing the costs of implementation strategies, it is also difficult, or even impossible, to compare different strategies or even implement them [14]. Accordingly, implementation studies should report the relevant costs of an implementation strategy, the sources of costs data, and how costs are calculated. Costs should include all costs from development to execution, such as staff, material, and training costs [12]. However, a previous systematic review showed that the quantity of economic evaluation in the field of implementation research is modest and called for more systematic and comprehensive reporting of costs in implementation research [12]. In economic evaluation of guideline implementation, there are three distinct stages: development of the guidelines, implementation of the guidelines, and treatment effects and costs as a consequence of behavior change. Systematic review of these cost brought out that costs were reported in a quarter of studies (27%), methodological quality was poor, and none of the included studies gave reasonable complete information of costs [18].

The above considerations are equally valid when de-implementation is concerned [19] as de-implementation processes are observed to be difficult and resource-intensive and the actual costs and subsequent savings are not well understood [3, 4]. A study [19] conceptualized the outcomes of de-implementation and recommended a clear distinction between the target of de-implementation and the strategies used. The recommendations included several aspects, such as potential cost savings due to decreased use of the target intervention, the costs of de-implementation strategies, the impacts on health care providers, and time, which should be considered when measuring the costs of de-implementation.

Since the aim of implementation and de-implementation is similar — to improve the quality of health care and effective use of resources — de-implementation strategies need to measure clinically relevant outcomes but also to analyze whether a strategy leads to a change in health care costs. The potential savings in health care costs as well as the costs of de-implementation strategy itself must be taken into account.

The aim of this systematic scoping review was to analyze how de-implementation studies have reported both the costs of de-implementation strategies and the impacts (estimated or measured) of de-implementation on health care costs.

## Methods

We used the PRISMA extension for scoping reviews (PRISMA-ScR) [20] to guide the conducting and reporting of this review (Additional file 1). This analysis of de-implementation costs and de-implementation impacts on health care costs was undertaken as part of a systematic scoping review of de-implementation randomized controlled trials (RCTs) [21]. This systematic scoping review was registered with Open Science Framework (OSF ueq32).

### Data sources and searches

Literature searches for these economic analyses are drawn from the registered systematic scoping review and are described in detail elsewhere [21]. We searched for de-implementation RCTs in the MEDLINE and Scopus databases up to May 24, 2021, without language or publication date limitations. The search strategy (Additional file 2) was developed in consultation with a medical information specialist (T. L.). We based our search on a previous scoping review identifying de-implementation-related terms [7] and modified it iteratively based on systematic reviews [22, 23]. We searched the reference lists of systematic reviews identified by our search to find additional potentially eligible articles. We also followed up protocols and post hoc analyses and added their main articles to the selection process.

### Study inclusion and exclusion criteria

The inclusion and exclusion criteria are described previously [21]. In brief, we included RCTs that aimed to reduce the use of a clinical practice. We included all de-implementation intervention types on any clinical practice and all target groups (patients, health care personnel, organizations, and citizens in general). We excluded articles on de-prescribing trials, because in our opinion the context is different (stopping a treatment already in use vs. not starting a treatment) [24]. We also excluded trials where one medical practice was used to de-implement another medical practice and trials where the reason to de-implementation was to reduce resource use (e.g., financial resources or clinical visits) [21].

### Risk of bias

To assess the quality of the included studies, we used a modified Cochrane risk-of-bias tool (RoB2.0) for randomized trials [25]. The process of modification is described in detail elsewhere [21]. This modified tool includes six criteria, judging studies to be at either high or low risk of bias (Additional file 3). The six criteria are as follows: (1) randomization procedure, (2) allocation concealment, (3) blinding of outcome collectors, (4) blinding of data analysts, (5) missing outcome data, and (6) imbalance of baseline characteristics. Four of the researchers conducted the quality assessment independently and in duplicate.

### Data collection and extraction strategy

Both independently and in duplicate, we used standardized forms with detailed instructions in identifying eligible articles (titles and abstract and full-text screening) and in data extraction. Disagreements were resolved through discussion and, if necessary, through consultation of a third investigator.

We collected the following data: (1) study characteristics (i.e., author(s), year, country of origin, sample size), (2) types and characteristics of interventions (i.e., intervention strategy, target groups of intervention), (3) characteristics of the practice of interest (i.e., target intervention, medical content area, medical settings), (4) outcomes of the study, (5) intervention efficacy, (6) costs of de-implementation (i.e., total costs, costs per unit), and (7) effect on health care costs (target group, size and direction of effect, and what was measured or estimated). The costs of de-implementation had to be reported in monetary form, and total or per unit cost were specified by the study authors. Data regarding costs of de-implementation and effect on health care costs are reported in this article; other outcomes are reported elsewhere [21].

### Data synthesis and analysis

We summarized the characteristics and details of de-implementation strategies and target population(s) and provided an overview on de-implementation costs. We extracted the costs in the reported currency and converted it into USD in 2021 value to facilitate comparability across all included studies. We changed the currency from EUR to USD, because more studies have used USD. We used a modified Effective Practice and Organisation of Care (EPOC) taxonomy [21] to categorize interventions and to analyze possible cost differences between different de-implementation strategies.

Finally, we provided an overview of the impact of de-implementation on health care costs. We reported the direction of the effects and cost allocations. We relied on the authors’ conclusion on the significance of the effect. We analyzed possible between-study differences in influence on health care costs. This was reported in various ways (monetary and qualitative).

We planned to do subgroup analyses based on (i) health care settings, (ii) target of intervention, (iii) health care financing, and (iv) country income groups. We assumed beforehand that the studies would be heterogeneous so a meta-analysis would not provide any added value.

We used summary statistics (i.e., frequencies and proportions) to describe study characteristics. We used nonparametric tests to analyze differences between outcomes of our interest. For statistical analyses, we used IBM® SPSS® version 28.0.1 (IBM Corp., Armonk, NY, USA), and all reported *P*-values less than 0.05 were considered statistically significant.

## Results

**Fig. 1:**
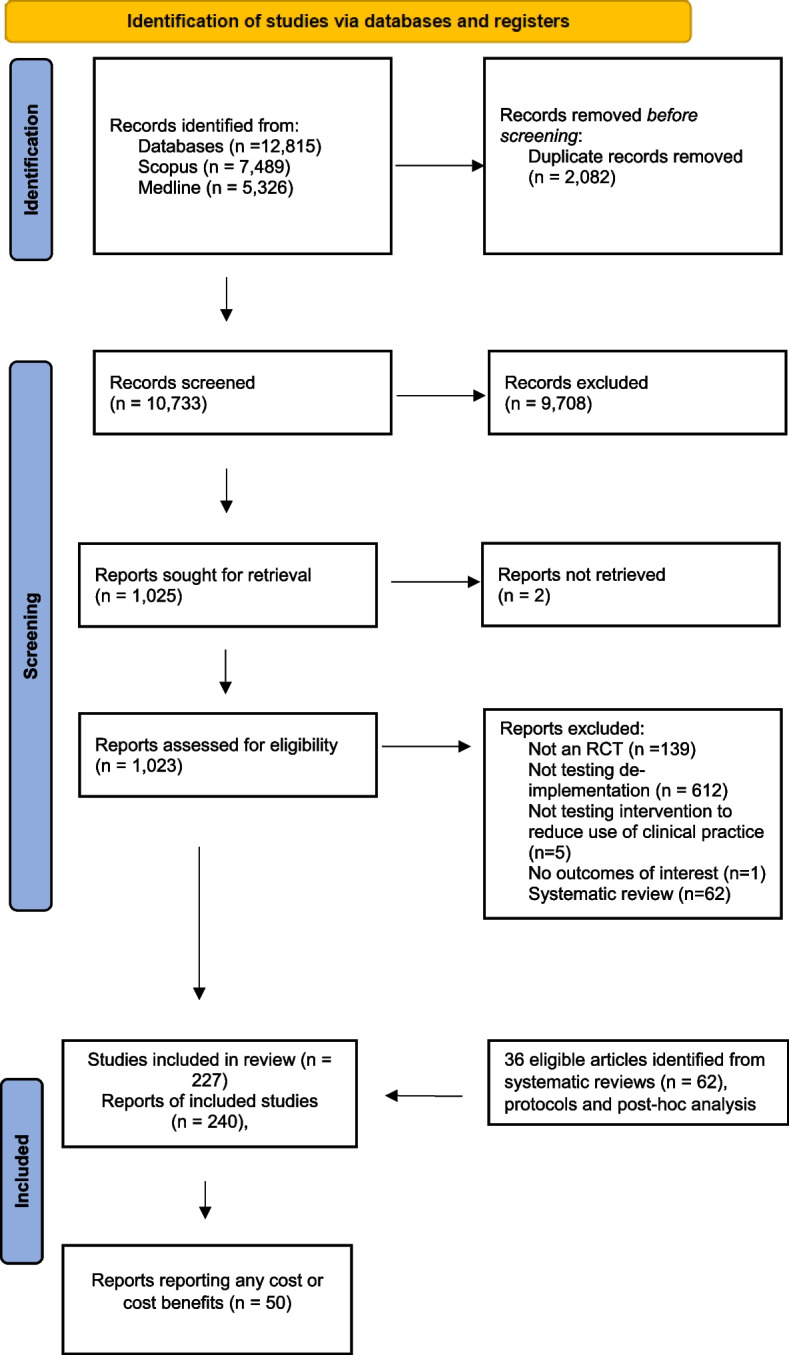
PRISMA flow diagram

Of the 12,815 articles identified in our search, we evaluated 1022 full-text articles. We included 227 RCTs, of which only 50 (22%) reported any costs or impact on health care costs. Figure 1 presents the PRISMA flow diagram, and a list all of included studies is found in Additional file 4.

The publication dates of the included articles ranged from 1982 to 2021, where half the articles dated after 2010. Most articles were from North America (*n* = 18, 36%) and Europe (*n* = 16, 32%). The majority of the studies (*n* = 41, 82%) were targeted to one type of professionals, and nine studies (18%) reported several target groups. Around two-thirds of the studies (*n* = 35, 70%) were conducted in primary care. The trials were aimed at reducing the use of drug treatments (*n* = 37, 74%), laboratory test (*n* = 8, 16%), or diagnostic imaging (*n* = 6, 12%). The studies used 16 different de-implementation strategies. Twenty-six used only one strategy, and twenty-four were multifaceted including two or more strategies. In all studies, the goal was to reduce use of a specific health care intervention. In 14 studies, an additional goal was replacing. The description of the characteristics of the included studies is shown in Table 1, and full characteristics are found in Additional file 5.

**Table 1:** Characteristics of the de-implementation interventions

Target	*N*	Cost information	Setting	*N*	Cost information
Physicians	35	13	Primary care outpatient	34	13
Patients	2		Primary care inpatient and outpatient	1	1
Nurses	2	1	Secondary or tertiary care outpatient	2	
Other health care providers	2		Secondary or tertiary care inpatient	12	3
Two or more target groups	9	4	Nursing home	1	1
**Medical content**^**a**^	***N***		**Clinical intervention**^**a**^	***N***	
Family/general practice	36	13	Drug treatment	37	15
Mixed, but not specified	8	3	Antibiotic use	26	
Emergency medicine	2		NSAID use	3	
General surgery	2	1	Overall prescribing	2	
Orthopedics	2	1	A combination of different drugs	6	
Anesthesiology	1		Laboratory tests	8	
Internal medicine	1		Diagnostic imaging	6	0
Obstetric	1		Blood transfusion	1	1
Oncology	1	1	Prevention	1	1
Pediatrics	1		Rehabilitation	1	1
Psychiatrics	1	1			
Pulmonary surgery	1	1			
Urology	1	1			
Vascular surgery	1	1			
**Number of used strategies**	***N***				
1	26	6			
2	13	5			
3	7	4			
4	4	3			

^a^Each trial could be categorized into several categories

### Risk of bias

Randomization was adequately generated in all studies. However, allocation concealment was not adequate in 10 studies (20%), 22 studies (44%) had missing data, and 20 (40%) had imbalance in baseline characteristics. Data collectors were blinded in 41 studies (82%) but data analysts in only two studies (4%) (Table 2).

**Table 2:**
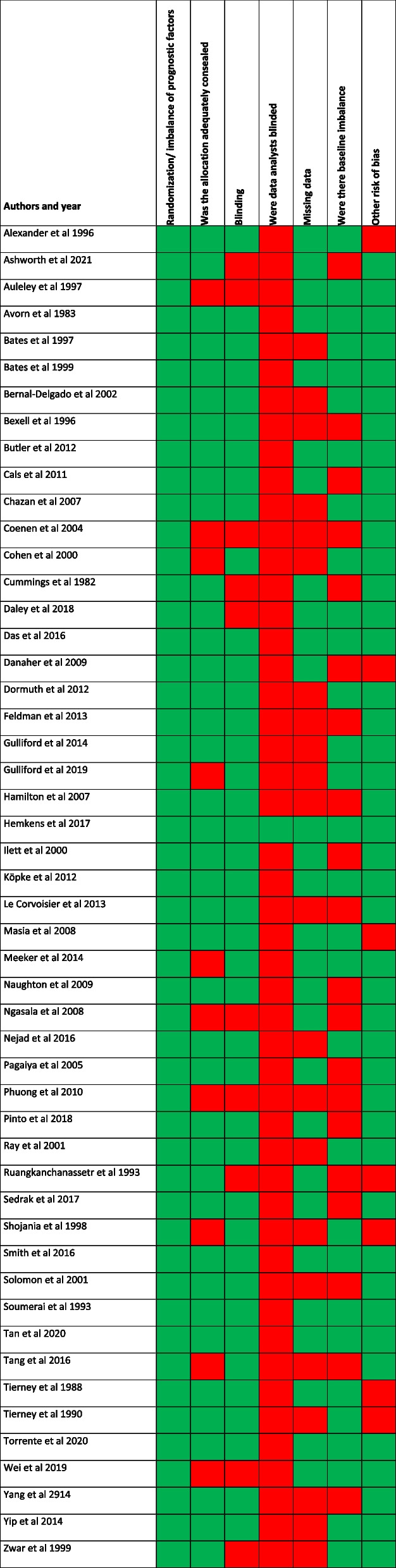
Risk-of-bias assessment

### De-implementation costs

The total costs of de-implementation intervention were reported in 13 studies (6%). These total costs varied considerably, the median being US $32,300 (range: US $616 to 747,000). Table 3 shows total costs converted to US dollars in 2021 value.

**Table 3:** De-implementation total costs and costs per unit, converted by the authors to USD 2021 value (on October 25, 2022)

Author and year	Target clinical intervention	Total costs in USD (2021 value)	Unit costs in USD (2021 value)
**Alexander et al. (1996) [**35**]**	**Rehabilitation**	**9710**^**b**^	
**Bexell et al. (1996) [**36**]**	**Drug treatment**	**6470**^**b**^	
**Dormuth et al. (2012) [**37**]**	**Drug treatment**	**118,000**^**b**^	
**Gulliford et al. (2019) [**38**]**	**Drug treatment, AB**	**747,000**^**a**^	
**Hemkens et al. (2017) [**39**]**	**Drug treatment**	**330,000**^**b**^	
**Köpke et al. (2012) [**40**]**	**Prevention**	**43,600**^**b**^	
**Ashworth et al. (2021) [**41**]**	**Drug treatment**	**7071**^**c**^	**10.2–65.2**^**3**^**/doctor**
**Butler et al. (2012) [**26**]**	**Drug treatment**	**163,000**^**1**^	**4950**^**1**^**/health care unit**
**Coenen et al. (2004) [**42**]**	**Drug treatment**	**6845**^**d**^	**280**^**4**^**/doctor**
**Gulliford et al. 2014) [**43**]**	**Drug treatment, AB**	**579,000**^**a**^	**0.46**^**1**^**/patient**
**Nejad et al. (2016) [**44**]**	**Drug treatment**	**616**^**b**^	**0.67**^**2**^**/doctor**
**Solomon et al. (2001) [**45**]**	**Drug treatment, AB**	**32,300**^**b**^	**32,300**^**2**^**/health care unit**
**Zwar et al. (1999) [**27**]**	**Drug treatment, AB**	**22,175**^**e**^	**284**^**5**^**/doctor**
**Avorn et al. (1983) [**46**]**	**Drug treatment**		**232**^**2**^**/doctor**
**Cals et al. (2011) [**47**]**	**Drug treatment**		**3.62**^**4**^**/patient**
**Das et al. (2016) [**48**]**	**Drug treatment**		**196**^**2**^**/health care provider**
**Soumerai et al. (1993) [**28**]**	**Blood transfusion**		**1290**^**2**^**/day**
**Wei et al. (2019) [**77**]**	**Drug treatment, AB**		**412**^**2**^**/health care unit**

References of currency conversions

^a^Lawrence H. Officer and Samuel H. Williamson, “Computing ‘Real Value’ Over Time With a Conversion Between U.K. Pounds and U.S. Dollars, 1791 to Present,” MeasuringWorth, 2022, www.measuringworth.com/exchange/

^b^Samuel H. Williamson, “Seven Ways to Compute the Relative Value of a U.S. Dollar Amount, 1790 to present,” Measuring Worth, 2022, https://www.measuringworth.com/calculators/uscompare/

^c^Lawrence H. Officer, “Exchange Rates Between the United States Dollar and Forty-one Currencies,” MeasuringWorth, 2022, http://www.measuringworth.com/exchangeglobal/

^d^4 first converted with reference c to USD, then with reference b to present value

^e^first converted with Diane Hutchinson and Florian Ploeckl, "Five Ways to Compute the Relative Value of Australian Amounts, 1828 to the Present", MeasuringWorth, 2022 https://www.measuringworth.com/calculators/australiacompare/ AUD in present value, then with reference c AUD to USD

The 13 studies (26%) that reported total costs used ten different de-implementation strategies. The most common strategies were educational materials (*n* = 9), audit and feedback (*n* = 7), educational meetings for individuals (*n* = 4), treatment algorithm (*n* = 3), educational meetings for groups (*n* = 3), and developing clinical practice guidelines (*n* = 2). The strategies used in one study included alerts, local consensus process, educational material for patients, and public intervention. Strategy combinations were diverse; many combined different educational strategies together. When using educational material in de-implementation, the median for total costs median was US $118,000 (range: US $6845 to 747,000). The total costs seemed higher in studies using educational materials than in studies not using such materials when not using it (Mann–Whitney test, *p* = 0.05). For other strategies, the total costs did not significantly differ between studies using vs. from not using each strategy (Mann–Whitney test, all *p* > 0.05).

In studies that used only one de-implementation strategy (*n* = 4, 31%), the median for total costs was US $8090 (range: US $616 to 32,300). In studies using two de-implementation strategies (*n* = 2, 15%), the median for total costs was US $224,000 (range: US $118,000 to 330,000), and with three or more strategies (*n* = 7, 54%), the median for total costs was US $43,600 (range US $6845 to 747,000).

Costs per unit were reported in 12 studies (5%). The most common unit was cost per physician, but also costs per health care provider, health care unit, day, and patient were reported (Table 3). In these studies, various de-implementation strategies were used. The most common were educational materials (*n* = 8), educational meetings for individuals (*n* = 6), and for groups (*n* = 5), audit and feedback (*n* = 5), and educational materials for patients (*n* = 2). Alerts, treatment algorithms, public intervention, and developing clinical practice guidelines were each used once. There were no differences in costs between using a given strategy and not using it (Mann–Whitney test, all *p* > 0.05).

Of the articles that reported total costs or costs per unit, 10 out of 18 (56%) offered at least some detailed information on the costs, but only four (22%) reported the exact costs. The most frequently reported types of costs were material costs, payment for trainers, and travel costs. In addition, postage, rent of premises, and loss of working hours were occasionally reported. Cost related to de-implementation intervention planning was rarely brought out. Information on costing methods was not mentioned in the articles. None of the articles separated costs related to the phases of de-implementation.

A meta-analysis was not possible due to the heterogeneity of the studies (e.g., the type and number of de-implementation strategies used). There were few studies in the pre-specified subgroups, so subgroup analyses were not appropriate.

### Impact on health care costs

The impact on health care costs was reported in 43 studies (19%). In most cases, the reports did not specify to whom the impact was targeted (*n* = 25, 58%). In four studies (9%), the impact was on patients’ own costs, whereas in 14 studies (33%), it was on health care provider’s costs. In 27 (63%) studies, health care costs decreased, whereas in 14 (33%), there was no change, and in two (5%) studies, the costs increased. The impact was targeted to medicine costs (*n* = 29, 67%), laboratory test costs (*n* = 8, 19%), total health care utilization costs (*n* = 3, 7%), diagnostic testing costs (*n* = 2, 5%), and radiography costs (*n* = 2, 5%).

Most of the articles (*n* = 32, 74%) have based their assessments on calculations on differences in costs between intervention and control group. In eight studies (19%), the authors have expanded the intervention group costs changes to large groups or for longer time. In one study [29], the authors have performed cost-effectiveness analyses, and in two studies, costs that were reported were costs changes during intervention period.

The two studies [30, 31] with increased costs had minor increases in costs allocated to patients. When the impact was allocated to the health care unit, the de-implementation either decreased costs (*n* = 12, 86%) or had no effect on costs (*n* = 2, 14%). In six studies, the authors estimated the impact on health care costs. De-implementation influenced laboratory test costs (*n* = 6), medicine costs (*n* = 5), diagnostic testing costs (*n* = 2), radiology costs (*n* = 2), and total health care expenditure per visit (*n* = 1). Table 4 shows the direction and size of the impact. The size of the impact was reported in different ways (Table 4).

**Table 4:** De-implementation impact on health care costs per allocated health care unit

Author + year	Target clinical intervention	Direction of impact	Size of impact	Size in US $ (2021 value)	Unit specified by study authors
**Bates et al. (1997) [**49**]**	**Diagnostic imaging, laboratory tests**	**Decreased**	**US $1.7 million**	**2.7 million**^**a**^	**Annual hospital charge**
**Bates et al. (1999) [**50**]**	**Laboratory tests**	**Decreased**	**US $35,000**	**54,300**^**a**^	**Annual**
**Daley et al. (2018) [**51**]**	**Drug treatment, AB**	**Decreased**	**US $14.94**	**15.0**^**a**^	**Per patient**
**Feldman et al. (2013) [**52**]**	**Laboratory tests**	**Decreased**	**US $436,115**	**507,000**^**a**^	**Per hospital/6 months**
**Shojania et al. (1998) [**53**]**	**Drug treatment, AB**	**Decreased**	**US $22,500**	**35,400**^**a**^	**Year**
**Soumerai et al. (1993) [**28**]**	**Blood transfusion**	**Decreased**	**US $3300**	**5670**^**a**^	**Per day of educational visit**
**Tierney et al. (1988) [**54**]**	**Diagnostic imaging, laboratory tests**	**Decreased**	**US $1.09**	**2.01**^**a**^	**Per patient**
**Tierney et al. (1990) [**55**]**	**Diagnostic imaging, laboratory tests**	**Decreased**	**US $6.68**	**11.20**^**a**^	**Per patient**
**Torrente et al. (2020) [**56**]**	**Drug treatment**	**Decreased**	**US $234,893**	**245,000**^**a**^	**Year in Argentina**
**Auleley et al. (1997) [**57**]**	**Diagnostic imaging**	**Decreased**	**130,000 FRF**	**35,400**^**b**^	**In 5 hospitals**
**Cohen et al. (2000) [**58**]**	**Drug treatment, AB**	**Decreased**	**8.9 FRF**	**1.52**^**b**^	**Per episode**
**Yip et al. (2014) [**59**]**	**Drug treatment, AB**	**Decreased**	**0.45–0.47 CNY**	**0.08**^**b**^	**Per visit**
**Tan et al. (2020) [**60**]**	**Diagnostic imaging**	**Unchanged**	**NA**		
**Sedrak et al. (2017) [**61**]**	**Laboratory tests**	**Unchanged**	**NA**		

References of currency conversions

^a^Samuel H. Williamson, “Seven Ways to Compute the Relative Value of a U.S. Dollar Amount, 1790 to present,” Measuring Worth, 2022, https://www.measuringworth.com/calculators/uscompare/

^b^first converted with Lawrence H. Officer, "Exchange Rates Between the United States Dollar and Forty-one Currencies,", MeasuringWorth, 2022, http://www.measuringworth.com/exchangeglobal/ to USD, then with reference **a** to present value

Of the 25 studies, which did not detail allocation of the impact, 14 (56%) reported a decrease in costs, whereas 11 (44%) reported no change (Table 5). The impact was calculated in twelve studies (48%) and estimated in five studies (20%). In the rest of studies, it was not possible to assess from the report whether the impact was calculated or estimated. In most of the studies (*n* = 20, 80%), the de-implementation mainly influenced the costs of medicine and laboratory tests. The change in reported health care cost varied between US $12.6 per patient to US $80.4 million per country. Of these 25 studies, 15 reported the impact in a monetary measure using different currencies (Table 5).

**Table 5:** De-implementation impact on health care costs in studies, not specifying to whom change was allocated

Author + year	Target clinical intervention	Direction of impact	Size of impact	Size in US $ (2021 value)
**Alexander et al. (1996) [**35**]**	**Rehabilitation**	**Decreased**	**US $319,000/101 patient**	**516,000/101 patient**^**b**^
**Avorn et al. (1983) [**46**]**	**Drug treatment**	**Decreased**	**US $19,740/year**	**45,800/year**^**b**^
**Chazan et al. (2007) [**62**]**	**Drug treatment, AB**	**Decreased**	**US $186/4-month season/patient**	**238/4-month season/patient**^**b**^
**Dormuth et al. (2012) [**37**]**	**Drug treatment**	**Decreased**	**US $465,000/2 years**	**550,000/2 years**^**b**^
**Meeker et al. (2014) [**63**]**	**Drug treatment, AB**	**Decreased**	**US $70.4 million/annual/country (USA)**	**80.4 million/annual/country (USA)**^**b**^
**Nejad et al. (2016) [**44**]**	**Drug treatment**	**Decreased**	**US $2000/3 months**	**2240/3 months**^**b**^
**Ray et al. (2001) [**64**]**	**Drug treatment**	**Unchanged**	**US $331/patient**	**491/patient**^**b**^
**Ilett et al. (2000) [**65**]**	**Drug treatment, AB**	**Decreased**	**16,130 AUD/3 months/56 GPs**	**21,815/3 months/56 GPs**^**d**^
**Zwar et al. (1999) [**27**]**	**Drug treatment, AB**	**Decreased**	**273 AUD/doctor**	**378/doctor $**^**d**^
**Butler et al. (2012) [**26**]**	**Drug treatment, AB**	**Decreased**	**830 GBP/year/practice**	**1410/year/practice**^**a**^
**Gulliford et al. (2019) [**38**]**	**Drug treatment, AB**	**Unchanged**	**51-GBP annual cost/patient**	**71.4/annual cost/patient**^**a**^
**Coenen et al. (2004) [**42**]**	**Drug treatment, AB**	**Decreased**	**7 EUR/patient**	**12.6/patient**^**c**^
**Danaher et al. (2009) [**66**]**	**Drug treatment, AB**	**Unchanged**	**58.67 EUR**	**127.4**^**c**^
**Le Corvoisier et al. (2013) [**67**]**	**Drug treatment, AB**	**Decreased**	**706 EUR**	**1073**^**c**^
**Masia et al. (2009) [**68**]**	**Drug treatment, AB**	**Unchanged**	**18 EUR/patient**	**31.26/patient**^**c**^
**Bernal-Delgado et al. (2002) [**69**]**	**Drug treatment**	**Unchanged**	**NA**	
**Cummings et al. (1982) [**70**]**	**Diagnostic imaging, laboratory tests**	**Decreased**	**NA**	
**Hamilton et al. (2007) [**71**]**	**Drug treatment**	**Unchanged**	**NA**	
**Naughton et al. (2009) [**29**]**	**Drug treatment, AB**	**Unchanged**	**NA**	
**Pagaiya et al. (2005) [**72**]**	**Drug treatment, AB and others**	**Decreased**	**NA**	
**Pinto et al. (2018) [**73**]**	**Drug treatment**	**Unchanged**	**NA**	
**Ruangkanchanasetr et al. (1993) [**74**]**	**Laboratory tests**	**Unchanged**	**NA**	
**Smith et al. (2016) [**75**]**	**Drug treatment, AB**	**Unchanged**	**NA**	
**Tang et al. (2016) [**76**]**	**Drug treatment, AB and injections**	**Decreased**	**NA**	
**Wei et al. (2019) [**77**]**	**Drug treatment, AB**	**Unchanged**	**NA**	
**Yang et al. (2014) [**78**]**	**Drug treatment, AB**	**Unchanged**	**NA**	

References of currency conversions

^a^Lawrence H. Officer and Samuel H. Williamson, “Computing ‘Real Value’ Over Time With a Conversion Between U.K. Pounds and U.S. Dollars, 1791 to Present,” Measuring Worth, 2022, www.measuringworth.com/exchange/

^b^Samuel H. Williamson, “Seven Ways to Compute the Relative Value of a U.S. Dollar Amount, 1790 to present,” Measuring Worth, 2022. https://www.measuringworth.com/calculators/uscompare/

^c^first converted with Lawrence H. Officer, "Exchange Rates Between the United States Dollar and Forty-one Currencies,", MeasuringWorth, 2022, http://www.measuringworth.com/exchangeglobal/ to usD, then with reference **b** to present value

^d^First converted with Diane Hutchinson and Florian Ploeckl, “Five Ways to Compute the Relative Value of Australian Amounts, 1828 to the Present,” Measuring Worth, 2022, https://www.measuringworth.com/calculators/australiacompare/ and then with Lawrence H. Officer, “Exchange Rates Between the United States Dollar and Forty-one Currencies,” MeasuringWorth, 2022, http://www.measuringworth.com/exchangeglobal/. All conversions have been made on 25th October 2022

The 43 studies that reported impact on health care costs used 15 different de-implementation strategies. The most common strategies were educational meetings for groups (*n* = 14, 33%), educational materials (*n* = 13, 30%), audit and feedback (*n* = 8, 19%), educational meetings for individuals (*n* = 6, 14%), treatment algorithm (*n* = 5, 12%), educational materials for patients (*n* = 5, 12%), and developing clinical practice guidelines (*n* = 3, 7%). Two studies used public release of performance data and patient-mediated interventions. The strategies used in one study included financial incentives for health care workers, local consensus process, local opinion leaders, managerial supervision, and routine patients-reported outcome measures.

Total costs of de-implementation and the impact on health care costs were reported in seven articles (14%), while unit costs and impact on health care costs were reported in five (10%) articles (Table 6). The articles by Zwar et al. [27] and Butler et al. [26] reported both total and unit costs, and the unit costs were in the same unit as the impact was reported. In two studies [27, 28], the intervention unit costs were less than their impact on health care costs. In the study by Butler et al. [26], the authors commented that their study decreased health care costs, but the intervention costs exceeded the savings.

**Table 6:** De-implementation costs and impact on health care costs in USD (converted by authors in October 2022)

Author + year	Target clinical intervention	Total costs in USD (2021 value)	Unit costs in USD (2021 value)	Impact size in USD (2021 value) and unit for which the impact is reported
**Alexander et al. (1996) [**35**]**	**Rehabilitation**	**9710**^**b**^	**NA**	**516,000**^**b**^**/101 patient**
**Avorn et al. (1983) [**46**]**	**Drug treatment**	**NA**	**232**^**b**^**/doctor**	**45,800**^**b**^**/year**
**Butler et al. (2012)** [26]	**Drug treatment, AB**	**163,000**^**a**^	**4950**^**a**^**/health care unit**	**1410**^**a**^**/health care unit**
**Coenen et al. (2004) [**42**]**	**Drug treatment, AB**	**6845**^**c**^	**280**^**c**^**/doctor**	**12.6**^**c**^**/patient**
**Dormuth et al. (2012) [**37**]**	**Drug treatment**	**118,000**^**b**^	**NA**	**550,000**^**b**^**/2 years**
**Gulliford et al. (2019) [**38**]**	**Drug treatment, AB**	**747,000**^**a**^	**NA**	**71.4**^**a**^**/patient**
**Nejad et al. (2016) [**44**]**	**Drug treatment**	**616**^**b**^	**NA**	**2240**^**b**^**/3 months**
**Soumerai et al. (1993)** [28]	**Blood transfusion**	**NA**	**1290**^**b**^**/day**	**5670**^**b**^**/day**
**Zwar et al. (1999)** [27]	**Drug treatment, AB**	**22,175**^**d**^	**284**^**d**^**/doctor**	**378**^**d**^**/doctor**

References of currency conversions

^a^Lawrence H. Officer and Samuel H. Williamson, “Computing ‘Real Value’ Over Time With a Conversion Between U.K. Pounds and U.S. Dollars, 1791 to Present,” Measuring Worth, 2022, http://measuringworht.com/exchange/

^b^Samuel H. Williamson, “Seven Ways to Compute the Relative Value of a U.S. Dollar Amount, 1790 to present,” Measuring Worth, 2022, https://www.measuringworth.com/calculators/uscompare/

^c^first converted with Lawrence H. Officer, "Exchange Rates Between the United States Dollar and Forty-one Currencies,", MeasuringWorth, 2022, www.measuringworth.com/exchangeglobal/ to USD, then with reference **b** to present value

^d^First converted with Diane Hutchinson and Florian Ploeckl, “Five Ways to Compute the Relative Value of Australian Amounts, 1828 to the Present,” Measuring Worth, 2022, www.measuringworth.com/calculators/australiacompare AUD in present value, and then with Lawrence H. Officer, “Exchange Rates Between the United States Dollar and Forty-one Currencies,” Measuring Worth, 2022, http://www.mearusingworth.com/exchangeglobal/. All conversions have been made on 25th October 2022

## Discussion

Even though de-implementation is often justified by emphasizing control of health care costs [5, 7, 32], our findings indicate that intervention costs or impact on health care costs was rarely reported in randomized trials of de-implementation. Even when costs were reported, the information on intervention costs or impact on health care costs was minimal. Costs related to data collection and analysis or de-implementation interventions planning were rarely brought out. We also found that methods for reporting intervention costs and impact on health care costs were heterogeneous, which obscures the relationship between costs and impact. Our results are similar to a previous systematic review in implementation research [13] that found aspects that were not adequately covered, such as project management costs, time costs for clinical time, and monitoring costs. A systematic review [18] on economic evaluations and cost analysis in guideline implementation found similar limitations in trial reporting. In all of the studies, the quality of cost information was limited, and only 27% of 235 studies reported any information on costs [18].

For economic evaluation, information on resource use, costs, time horizons, health outcomes, or the consequences of interventions are necessary [33]. Incomplete cost information on de-implementation interventions does not allow economic evaluation or, at worst, may lead to distorted conclusions. The lack of cost information has been identified as a barrier to implementation [12, 14]. De-implementation requires sufficient financial, technical, and human resources [34]. The lack of cost information makes it impossible to evaluate the costs in a systematic way or to basing decision-making on this information. Knowledge-based decisions become possible only when intervention costs and impact on health care costs are both known.

To improve the utilization of de-implementation research, economic evaluation should be planned along with the research. Subsequently, the studies should report precise monetary costs of de-implementation strategies and their impact on health care costs. When reporting costs, general considerations should be taken into account: (i) give detailed and reasoned values, (ii) separate included costs, (iii) provide the time horizon when the costs are applicable, and (iv) break down the planning and acting phase costs of the de-implementation process.

### Strengths and limitations

Our highly sensitive literature search used a wide variety of de-implementation terms. However, due to heterogeneous indexing of de-implementation studies, it is possible that we may have missed relevant articles.

A strength of our article is that we searched for cost information and de-implementation impact information from the full text of articles, which noticeably increased the number of included articles — as the impact on health care costs tended to be reported in the full text, not in the abstract.

We restricted our study to RCTs, which may be seen as a limitation. Since many de-implementation projects have likely not included randomized control groups, we missed economic information from these studies. However, the efficacy of interventions should be studied in RCT settings, and thus, we believe that our decision to exclude other study designs is justified.

This review was made alongside with another review, which may have restricted the number of included studies. We excluded studies where one medical practice was used to de-implement another, because these often focus on implementation not on de-implementation. We focused on de-implementation of low-value care, and therefore, excluded articles were the reason for de-implementation which was cutting resource use. Both these restrictions may have excluded some articles where cost information could have been given. However, we believe that our careful selection of articles in the full-text phase has prevented this. Our perspective was to find out how costs are brought out in de-implementation studies, so the search was made on that view. It could be a limitation, and some studies with costs may have been missed. To avoid this, we searched also the references of included studies to find other articles on same studies. Using the approach that Vale has used, it may lead to a different result.

## Conclusion

A vast majority of de-implementation trials have failed to report any intervention costs or impacts on health care costs. In studies that do include cost information, typically only nonnumerical information on economics impacts is reported, and direct costs of de-implementation strategies are excluded. To advance the field, researchers should consider economic aspects and include health economists when planning research. De-implementation interventions are often complex and resource intensive, and cost information is essential for effective health policymaking.

### Supplementary Information

**Additional file 1.** PRISMA-ScR checklist.

**Additional file 2.** Search strategy.

**Additional file 3.** RoB tool.

**Additional file 4.** List of included studies.

**Additional file 5.** Characteristics of included studies.

## Data Availability

The datasets generated during and/or analyzed during the current study are available from the corresponding author on reasonable request.

## Abbreviations

PRISMA-ScR: Prisma extension for scoping review
RCT: Randomized controlled trial
RoB: Risk of bias
USD: US dollars
EPOC: Effective practice and organization of care taxonomy
FRF: French franc
CNY: Renminbi
AUD: Australian dollars
GBP: Great Britain pounds
AB: Antibiotics
QHES: Quality of health economics studies
