# Supplementary material for: Reporting of costs and economic impacts in randomized trials of de-implementation interventions for low-value care: a systematic scoping review
Source: Implement Sci. 2023 Aug 21;18:36. doi: 10.1186/s13012-023-01290-3 (PMC10440866; doi:10.1186/s13012-023-01290-3)
Supplement: Supplementary file 2 — Additional file 2. Search strategy. [file 13012_2023_1290_MOESM2_ESM.docx]

Additional file 2

Search strategy

At first, we used the most important de-implementation related terms listed by Niven et al. Due to heterogenous indexing we compared it to number of articles we found from systematic reviews on de-implementation RCTs. The number of articles was relatively low, so we updated the search strategy by with terms that were found in articles not included in search strategy with Niven et al. terms. We avoided using terms that were clearly related to specific medical content areas or specific medical treatments or tests. Although it might lead to missing some articles in the search, including those specific term could bias the results towards those medical content areas or treatments.

**Scopus**

Search history 26.5.2021

( ( TITLE-ABS-KEY ( "randomized controlled trial" ) ) OR ( TITLE-ABS-KEY ( "controlled clinical trial" ) ) OR ( ABS ( randomized OR placebo OR randomly ) ) OR ( TITLE ( trial ) ) ) AND ( ( ( TITLE-ABS-KEY ( abandon* OR de-list* OR dis-invest* OR discontinu* OR dis-continu* OR decommiss* OR de-commiss* OR deadopt* OR de-adopt* OR de-implement* OR deimplement* OR reduc* OR remov* OR stop* OR relinquish* OR disadopt* OR disinvest* OR de-prescri* OR deprescri* ) ) OR ( TITLE-ABS-KEY ( ( decreas* W/3 "use" ) OR ( declin* W/3 "use" ) OR ( drop* W/3 "use" ) OR ( decreas* W/3 rate* ) ) ) OR ( TITLE-ABS-KEY ( withdraw* OR replac* OR reallocat* OR re-allocat* ) ) OR ( TITLE-ABS-KEY ( "change* in use" OR "change* in practice*" ) ) OR ( TITLE-ABS-KEY ( improv* W/4 "use" ) ) OR ( TITLE-ABS-KEY ( reduc* W/4 "use" ) ) OR ( TITLE-ABS-KEY ( change* W/4 "use" OR change* W/3 practice* ) ) ) AND ( ( TITLE-ABS-KEY ( inappropriate W/3 prescri* ) ) OR ( TITLE-ABS-KEY ( "Health Service*" W/1 misus* ) ) OR ( TITLE-ABS-KEY ( low-value ) ) OR ( TITLE-ABS-KEY ( ( overutili* OR overus* OR overdiagnos* OR overtreat* OR overmedicat* OR overprescrib* ) ) ) OR ( TITLE-ABS-KEY ( ( unnecessary OR ineffective OR useless OR inefficient OR valueless ) W/1 ( care OR usage OR utilisation OR utilization OR treatment* OR intervention* OR practice* OR procedure OR drug* OR therap* OR technolog* OR device* OR surg* OR test* OR lab* OR imaging ) ) ) OR ( TITLE-ABS-KEY ( "unnecessary use" ) ) OR ( TITLE-ABS-KEY ( "INAPPROPRIATE use" ) ) OR ( TITLE-ABS-KEY ( obsolete ) ) OR ( TITLE-ABS-KEY ( contradict* OR refute* OR reassess* OR re-assess* OR re-apprais* OR reapprais* OR revers ) ) OR ( TITLE-ABS-KEY ( "Guideline Adherence" ) ) ) )

( ( TITLE-ABS ( "randomized controlled trial" ) ) OR ( TITLE-ABS ( "controlled clinical trial" ) ) OR ( TITLE-ABS ( randomized ) ) OR ( TITLE-ABS ( placebo ) ) OR ( TITLE-ABS ( randomly ) ) OR ( TITLE ( trial ) ) ) AND ( ( TITLE-ABS-KEY ( "Unnecessary Procedure*" ) ) OR ( TITLE-ABS-KEY ( "prescription rate*" ) ) OR ( TITLE-ABS-KEY ( "Guideline adherence" ) ) OR ( TITLE-ABS-KEY ( "too much medicine" ) ) OR ( TITLE-ABS-KEY ( "choosing wisely" ) ) OR ( TITLE-ABS-KEY ( "do not do" ) ) OR ( TITLE-ABS-KEY ( deprescription* ) ) OR ( TITLE-ABS-KEY ( guideline* W/3 implementation* ) ) OR ( TITLE-ABS-KEY ( reduc* W/4 prescri* ) ) OR ( TITLE-ABS-KEY ( unnecessary W/4 "use" ) ) OR ( TITLE-ABS-KEY ( unnecessary W/4 prescri* ) ) OR ( TITLE-ABS-KEY ( "Inappropriate prescri*" ) ) OR ( TITLE-ABS-KEY ( decreas* W/3 referrals ) ) )

**Medline**

Database: Ovid MEDLINE(R) ALL <1946 to May 24, 2021>

Search Strategy:

--------------------------------------------------------------------------------

1 Unnecessary Procedures/

2 prescription rate*.tw.

3 Guideline adherence.tw.

4 too much medicine.tw.

5 choosing wisely.tw.

6 "do not do".tw.

7 deprescriptions/

8 (guideline* adj3 implementation*).tw.

9 Practice Patterns, Physicians'/sn, st

10 Physicians, Family/st, sn [Standards, Statistics & Numerical Data]

11 exp Drug Utilization/sn [Statistics & Numerical Data]

12 randomized controlled trial.pt.

13 controlled clinical trial.pt.

14 randomized.ab.

15 placebo.ab.

16 clinical trials as topic.sh.

17 randomly.ab.

18 trial.ti.

19 12 or 13 or 14 or 15 or 16 or 17 or 18

20 exp animals/ not humans.sh.

21 19 not 20

22 1 or 2 or 3 or 4 or 5 or 6 or 7 or 9 or 10 or 11

23 21 and 22

24 (reduc* adj4 prescri*).tw.

25 (unnecessary adj4 "use").mp. [mp=title, abstract, original title, name of substance word, subject heading word, floating sub-heading word, keyword heading word, organism supplementary concept word, protocol supplementary concept word, rare disease supplementary concept word, unique identifier, synonyms]

26 (unnecessary adj4 prescri*).tw.

27 Inappropriate Prescribing/

28 (decreas* adj3 referrals).tw.

29 inappropriate prescri*.tw.

30 24 or 25 or 26 or 27 or 28 or 29

31 21 and 30

32 23 or 31

Database: Ovid MEDLINE(R) ALL <1946 to May 24, 2021>

Search Strategy:

--------------------------------------------------------------------------------

1 exp Health Services Misuse/

2 Inappropriate Prescribing/

3 low-value.tw.

4 (overutili* or overus* or overdiagnos* or overtreat* or overmedicat* or overprescrib*).tw.

5 ((unnecessary or ineffective or useless or inefficient or valueless) adj (care or usage or utilisation or utilization or treatment* or intervention* or practice* or procedure or drug* or therap* or technolog* or device* or surg* or test* or lab* or imaging)).tw.

6 "unnecessary use".tw.

7 Inappropriate Prescri*.tw.

8 "INAPPROPRIATE use".tw.

9 obsolete.tw.

10 (contradict* or refute* or reassess* or re-assess* or re-apprais* or reapprais* or revers).tw.

11 exp Guideline Adherence/

12 1 or 2 or 3 or 4 or 5 or 6 or 7 or 8 or 9

13 (abandon* or de-list* or dis-invest* or discontinu* or dis-continu* or decommiss* or de-commiss* or deadopt* or de-adopt* or de-implement* or deimplement* or reduc* or remov* or stop* or relinquish* or disadopt* or DISINVEST* or de-prescri* or Deprescri*).tw.

14 ((decreas* adj3 "use") or (declin* adj3 "use") or (drop* adj3 "use") or (decreas* adj3 rate*)).mp.

15 (withdraw* or replac* or reallocat* or re-allocat*).tw.

16 ("change* in use" or "change* in practice*").tw.

17 (improv* adj4 "use").mp.

18 ((change adj4 "use") or "change* in practice").mp.

19 (reduc* adj4 "use").tw.

20 13 or 14 or 16 or 17 or 18 or 19

21 12 and 20

22 randomized controlled trial.pt.

23 controlled clinical trial.pt.

24 randomized.ab.

25 placebo.ab.

26 clinical trials as topic.sh.

27 randomly.ab.

28 trial.ti.

29 22 or 23 or 24 or 25 or 26 or 27 or 28

30 exp animals/ not humans.sh.

31 29 not 30

32 21 and 31
