# Supplementary material for: Reporting of costs and economic impacts in randomized trials of de-implementation interventions for low-value care: a systematic scoping review
Source: Implement Sci. 2023 Aug 21;18:36. doi: 10.1186/s13012-023-01290-3 (PMC10440866; doi:10.1186/s13012-023-01290-3)
Supplement: Supplementary file 3 — Additional file 3. RoB tool. [file 13012_2023_1290_MOESM3_ESM.docx]

Additional file 3

Risk of Bias tool

1. **Randomization/imbalance of prognostic factors**
   1. **Was the allocation sequence adequately generated?**

Definitely yes Probably yes Probably no Definitely no

(low risk of bias) (high risk of bias)

The use of a random component should be sufficient for adequate sequence generation. This could be achieved by allocating interventions using methods such as repeated coin-tossing, throwing dice or dealing previously shuffled cards. If the allocation was by telephone or Internet, the randomization was done through a computer system.

Examples of low risk of bias (“definitely yes”): Referring to a random number table; Using a computer random number generator; Coin tossing; Shuffling cards or envelopes; Throwing dice; Drawing of lots; Minimization with or without a random element.

Examples of high risk of bias (“definitely no”): Sequence generated by odd or even date of birth; Sequence generated by some rule based on date (or day) of admission; Sequence generated by some rule based on hospital or clinic record number; Allocation by judgement of the clinician; Allocation by preference of the participant; Allocation based on the results of a laboratory test or a series of tests; Allocation by availability of the intervention.

If they say “randomized” and give no more information regarding sequence generation, the process was probably low risk of bias, so, answer “Probably yes”.

- 1. **Was the allocation adequately concealed?**

Definitely yes Probably yes Probably no Definitely no

(low risk of bias) (high risk of bias)

In randomized trials, allocation concealment strategies hide the method of sorting trial participants into treatment groups so that this knowledge cannot be exploited. Adequate allocation concealment serves to prevent trial investigators/recruiters from choosing treatment allocations for individuals/patients. Studies with poor allocation concealment (or none at all) are prone to selection bias. Trials where participants are recruited before randomization are low risk of bias. Trials where participants are recruited between randomization and the beginning of the intervention are usually high risk of bias.

If there are multiple levels of recruitment, consider allocation concealment on the lowest level. For instance, educational intervention targeted to physicians: clusters are health care centers, physicians are participants to whom the educational intervention was targeted and patients were participants to whom the medical intervention was targeted. If patients are recruited after the randomisation we will consider the study as high risk of bias. An exception is, however, if all patients from the physician were analysed or otherwise it was impossible for the physician to decide which patients were included. In this case we will consider study as low risk of bias.

Examples of low risk of bias (“definitely yes”):

1. i) the unit of allocation was by patient or episode of care AND ii) there was some form of centralized randomization scheme and on-site computer system OR sealed opaque envelopes were used.
2. i) the unit of allocation was by institution, team or professional AND ii) less than 5% were recruited (at the lowest level) after the randomization
3. i) the unit of allocation was by institution, team or professional AND ii) Lowest level of recruitment was conducted after the randomization AND iii) recruiter(s) were blinded to study groups OR all participants were recruited

Examples of low risk of bias (“probably yes”):

1. i) the unit of allocation was by institution, team or professional AND ii) less than 10% were recruited (at the lowest level) after the randomization

Examples of high risk of bias (“probably no”):

1. the unit of allocation was by patient or episode of care and there was some form of centralized randomization scheme and allocation concealment was not reported.
2. the unit of allocation was by institution, team or professional and recruitment of study participants and It’s unclear how many participants were recruited after the randomization.

Examples of high risk of bias (“definitely no”):

1. the unit of allocation was by patient or episode of care AND Using an open random allocation schedule (e.g. a list of random numbers) OR Assignment envelopes were used without appropriate safeguards (e.g. if envelopes were unsealed or non-opaque or not sequentially numbered) OR Alternation or rotation OR Date of birth OR Case record number OR Any other explicitly unconcealed procedure.
2. i) the unit of allocation was by institution, team or professional AND ii) allocation was not performed for all at the start of the study (over 10% recruitment of study participants after the randomization)

1. **Blinding. Was knowledge of the allocated interventions adequately prevented?**
   1. **Were data collectors/outcome assessors blinded?**

Definitely yes Probably yes Probably no Definitely no

(low risk of bias) (high risk of bias)

Low risk of bias (“definitely yes”):

Data were collected from medical records or other database and blinding of data collectors was reported.

In other situations, if data collectors did not know to which group study participants belong.

Low risk of bias (“probably yes”):

Data were collected from medical records or other database and blinding of data collectors was not reported.

High risk of bias (“probably no”):

In trials where data were not collected from medical records or other database, if blinding was not reported.

High risk of bias (“definitely no”):

Data were collected from medical records or other database and it was somehow stated that data collectors were not blinded or non-blinding is obvious other way.

In other situations, if non-blinding was reported.

If there were several ways of collecting the data, assess the way that primary outcome(s) was collected. If there was no reported primary outcome(s), primarily assess regarding the outcome of prevalence of low-value care/total volume of care was collected and secondarily assess regarding the outcome of intention/perception to reduce low-value care.

- 1. **Were data analysts blinded?**

Definitely yes Probably yes Probably no Definitely no

(low risk of bias) (high risk of bias)

Answer “Probably no” unless there is some specific indication implying that data analysts were blinded.

1. **Missing data (by primary outcome):**

Definitely yes Probably yes Probably no Definitely no

(high risk of bias) (low risk of bias)

Thresholds for risk of bias judgements. Respond:

“Definitely no”:

Less than 5% of the primary outcome data is missing (low risk of bias)

“Probably no”:

At least 5% but less than 10% of the outcome data is missing (low risk of bias)

“Probably yes”:

At least 10% but less than 20% of the outcome data is missing (high risk of bias)

“Definitely yes”:

At least 20% of the outcome data is missing (high risk of Bias)

Preferably use the primary outcome for assessment of missing data/drop outs. If there is no primary outcome, i) primarily use the outcome(s) measuring total volume of care/prevalence of low-value care; and ii) secondarily outcome(s) measuring intention/perception/willingness to reduce low-value care.

If there is one primary outcomes, use the proportion of missing data for judgment. If there are several primary outcomes (or if there is no primary outcome and other outcome types are used), use median values of missing outcome data for outcome category.

When judging missing data, please, remember to consider all levels of recruitment where missing data/drop outs are possible. If drop outs are highly unlikely in every level, answer “probably no” (in individually randomised trials this means only one level).

Drop outs are considered as highly unlikely when recruitment/allocation, intervention and outcome measurement happens within the same encounter.

For example, **i)** patient may be recruited, allocated, given the intervention and outcome assessed/measured within the same visit to the health care center or **ii)** physician may be recruited, allocated, intervention given and outcome measured within the same encounter (e.g. authors use survey to measure willingness to reduce use of low-value care after educational intervention and everything from allocation to measurement is done within the same encounter). In these situations, record as “probably no”.

Drop outs that are included in the analysis (intention to treat principle) are not considered as missing outcome data. Sometimes authors use “intention to treat principle” in the analysis (and include participants that did drop out but could still be followed up to measure the outcomes), but there is still drop outs with missing outcome data that they can’t include in the analysis.

1. **Were there baseline imbalances at the start of the trial?**

Definitely yes Probably yes Probably no Definitely no

(high risk of bias) (low risk of bias)

Consider baseline estimates of (primary) outcome and age/work experience between intervention and control groups.

Thresholds for risk of bias judgements. Respond:

“definitely no”: Relative difference (compared to control group) is less than 5% (low risk of bias)

“probably no”: Relative difference (compared to control group) at least 5% but less than 10%

“probably yes”: Relative difference (compared to control group) at least 10% but less than 20%

“definitely yes”: Relative difference (compared to control group) at least 20% (high risk of bias)

Examples of relative difference:

if use of antibiotics 80% in intervention and 60% in control group relative difference here is not 20% but it is 33%

if median age is 68 years in intervention and 60 years in control group, relative difference is here 13%

If not reported, answer “probably yes”. An exception is, however, when cluster unit is team or institution AND the number of clusters is over 20 OR if trial is individually randomized/cluster unit individual professional AND number of clusters/individuals is over 100. In this case answer “probably no”.

If there are more than 1 primary outcome, we will use the median value (of proportion of missing data). If no outcome is clearly reported as primary outcome, we will use the median value (of proportion of missing data) of all outcomes that measure prevalence of low value care use. If there are no outcomes reported measuring prevalence of low value care use, we will use the median value (of proportion of missing data) of outcomes that measure intention/perception to reduce low-value care.

1. **Other risk of bias?**
   1. **choose at least one**
      1. **Contamination**
      2. **Selective reporting**
      3. **Other**
      4. **No**
