# Supplementary material for: Reporting of costs and economic impacts in randomized trials of de-implementation interventions for low-value care: a systematic scoping review
Source: Implement Sci. 2023 Aug 21;18:36. doi: 10.1186/s13012-023-01290-3 (PMC10440866; doi:10.1186/s13012-023-01290-3)
Supplement: Supplementary file 4 — Additional file 4. List of included studies. [file 13012_2023_1290_MOESM4_ESM.docx]

Additional file 4

List of included studies

**Alexander E, Weingarten S, Mohsenifar Z**. Clinical Strategies to Reduce Utilization of Chest Physioterapy Without Compromising Patient Care. Chest 1996;110:430-32

**Ashworth N, Kain N, Wiebe D, Hernandez-Ceron N, Jess E, Mazurek** K. Reducing prescribing of benzodiazepines in older adults: a comparison of four physician -focused interventions by a medical regulatory authority. BMC Fam Pract 2021;22:68. <https://doi.org/10.1186/s12875-021-01415-x>

**Auleley G-R, Ravaud P, Giraudeau B, Kerboull L, Nizard R, Masssin** P. et al. Implementation of the Ottawa Ankle Rules in France. JAMA 1997;277:1935-1939

**Avorn J, Soumerai SB**. Improving Drug-Therapy Decisions through Educational Outreach, a Randomized Controlled Trial of Academically Based “Detailing”. N Engl J Med 1983;308:1457-63

**Bates DW, Kuperman GJ, Jha A, Teich JM, Orav EJ, Ma’luf N, et al**. Does the Computerized Display of Changes Affect Inpatient Ancillary Test Utilization? Arch Intern Med. 1997;157:2501-2508

**Bates DW, Kupermann GJ, Rittenberg E, Teich JM, Fiskio J, Ma’luf N, et a**l. A randomized Trial of a Computer-based Intervention to Reduce Utilization of Redundant Laboratory Tests. Am J Med. 1999;106: 144-150.

**Bernall-Delgado E, Galeote-Mayor M, Pradas-Arnal F, Moreno-Peiró S**. Evidence based educational outreach visits: effects on prescriptions of non-steroidal anti-inflammatory drugs. J. Epidemiol Community Health 2002;56:653-658.

**Bexell A, Lwando E, von Hofsten B, Tembo S, Eriksson B, Diwan VK**. Improving Drug Use through Continuing Education: A Randomized Controlled Trial in Zambia. H Clin Epidemiol 1996;49(3):355-357

**Butler CC, Simpson SA, Dunstan F, Rollnick S, Cohen D, Gillespie D, et a**l. Effectiveness of multifaceted educational programme to reduce antibiotic dispensing in primary care: practice based randomised controlled trial. BMJ 2012;344:d8173.  <https://doi.org/10.1136/bmj.d8173>

**Cals JWL, Ament AJHA, Hood K, Butler CC, Hopstaken RM, Wassink G, et al**. C-reactive protein point of care testing and physician communication skills training for lower respiratory tract infections in general practice: economic evaluation of a cluster randomized trial. J of Evaluation in Clinical Practice 2011; 17:1059-1069. <https://doi.org/10.1111/j.1365-2753.2010.01472.x>

**Chazan B, Ben Zur Turjeman R, Frost Y, Besharat B, Tabenkin H, Stainberg A, et al**. Antibiotic Consumption Succesfully Reduced by a Community Intervention Program. IMAJ 2007;9:16-20

**Coenen S, Van Royen P, Michiels B, Denekens** J. Optimizing antibiotic prescribing for acute cough in general practice: a cluster-randomized controlled trial. Journal of Antimicrobial Chemotherapy 2004;54:661-672. <https://doi.org/10.1093/jac/dkh374>

**Cohen R, Allart FA, Callens A, Menn S, Urbinelli R, Roden** A. Evaluation médico-économique d’une intervention éducative pour l’optimisation du treatment des rhinopharyngites aiguës non compliquées de l’enfant en pratique ville. Méd Mal Infect 2000;30:691-8.

**Cummings KM, Frisof KB, Long MJ, Hrynkiewich G**. The Efects of Price Information on Physicians’ Test-Ordering Behavior. Medical Care 1982; XX (3): 293- 301

**Danaher PJ, Milazzo NA, Kerr KJ, Lagasse CA, Lane JW**. The Antibiotic Support Team. A Successful Educational Approach to Antibiotic Stewardship. Military Medicine, 2009; 174(2):201.

**Daley P, Garcia D, Inayatullah R, Penney C, Boyd S**. Modified Reporting of Positive Urine Cultures to Reduce Inappropriate Treatment of Asymptomatic Bacteriuria Among Nonpregnant, Noncatheterized Inpatients: A Randomized Controlled Trial. Infect Control Hosp Epidemiol 2018;39:814-819. <https://doi.org/10.1017/ice.2018.100>

**Das J, Chowdhury A, Hussam R, Banerjee AV**. The impact of training informal health care providers in India: A randomized controlled trial. Science 2016, 354, aaf7384. DOI: [10.1126/science.aaf7384](https://doi.org/10.1126/science.aaf7384)

**Dormuth CR, Carney G, Taylor S, Bassett K, Maclure MA**. Rondomized Trial Assessing the Impact of a Personal Printed Feedback Portrait on Statin Prescribing in Primary Care. J of Continuing Education in the Health Professions 2012;32(3):153-162

**Feldman LS, Shihab HM, Thiemann D, Yeh HC, Ardolino M, Mandell S, et al**. Impact of Providing Fee Data on Laboratory Test Ordering. A Controlled Clinical Trial. JAMA Intern Med. 2013;273(10):903-908. doi:10.1001/jamainternmed.2013.232

**Gulliford MC, van Staa T, Dregan A, McDermot L, McCann G, Ashworth M, et al**. Electronic Health Records for Intervention Research: A Cluster Randomized Trial to Reduce Antibiotic Prescribing in Primary Care (eCRT Study). Ann Fam Med 2014;344-351. DOI: [10.1370/afm.1659](https://doi.org/10.1370/afm.1659)

**Gulliford MC, Juszczyk D, Prevost AT, Soames J, McDermott L, Sultana K, et al**. Electronically-delivered, multi-component intervention fro antimicrobial stewardship in primary care. Cluster randomized controlled trial (REDUCE trial) and cohort study of safety outcomes. BMJ (Clinical research ed.)2019;364:1236.

**Hamilton W, Russell D, Stabb C, Seamark D, Campion-Smith C, Britten N**. The effect of patient self-completion agenda forms on prescribing and adherence in general practice: a randomized controlled trial. Family Practice 2007;24:77-83. <https://doi.org/10.1093/fampra/cml057>

**Hemkens LG, Saccilotto R, Reyes SL, Glinz D, Zumbrunn T, Grolimund O, et al**. Personalized Prescription Feedback Using Routinely Collected Data to Reduce Antibiotic Use in Priamry Care.A Randomized Clinical Trial. JAMA Intern Med. 2017;177(2):176-183. doi:10.1001/jamainternmed.2016.8040

**Ilett KF, Johnson S, Greenhill G, Mullen L, Brockis J, Golledge CL, et al**. Modification of general practitioner prescribing of antibioitics be use of a therapeutics adviser 8academic detailer). J Clin Pharmacol 1999;49:168-173

**Köpke S. Mühlhauser I. Gerlach A. Haut A. Haastert B. Möhler R et al**. Effect of a Guideline-Based Multicomponent Intervention on Use of Physical Restraints in Nursing Homes. A Randomized Controlled Trial. JAMA 2012;307(20):2177-2184.

**Le Corvoisier P, Renard V, Roudot-Thoraval F, Cazalens T, Veerabudun K, Canoui-Poitrine F, et al**. Long-term effects of an educational seminar on antibiotic prescribing by GPs: a randomised controlled trial. Br J Gen Pract 2013; 63 (612):e455-64. DOI: [10.3399/bjgp13X669176](https://doi.org/10.3399/bjgp13x669176)

**Masia M, Matoses C, Padilla S, Murcia A, Sánchez V, Romero I, et al**. Limited efficacy of a nonresticted intervention on antimicrobial prescription of commonly used antibiotics in the hospital setting: results of a randomized controlled trail. Eur J Clin Microbiol Infect Dis 2008;27:597-605. DOI: [10.1007/s10096-008-0482-x](https://doi.org/10.1007/s10096-008-0482-x)

**Meeker D. Knight TK, Friedberg MW, Linder JA, Goldstein NJ, Fox GR, et al**. Nudging Guideline-Concordant Antibiotic Prescribing. A Randomized Clinical Trial. JAMA Intern Med. 2014;174(39:425-431. DOI: [10.1001/jamainternmed.2013.14191](https://doi.org/10.1001/jamainternmed.2013.14191)

**Ngasala B, Mubi M, Warsame M, Petzold MG, Massele AY, Gustafsson LL, et a**l. Impact of training in clinical and microscopy diagnosis of childhood malaria on antimalarial drug prescription and health outcome at primary health care level in Tanzania: A randomized controlled trial. Malaria Journal 2008;7:199. <https://doi.org/10.1186/1475-2875-7-199>

**Naughton C, Feely J, Bennett K**. A RCT evaluating the effectiveness and cost-effectiveness of academic detailing versus postal prescribing feedback in changing GP antibiotic prescribing. J of evaluation in Clinical Practice 2009;15:807-812. <https://doi.org/10.1111/j.1365-2753.2008.01099.x>

**Nejad AS, Noori MRF, Haghdoost AA, Bahaadinbeigy K, Abu-Hanna A, Eslami S**. The Effect of registry-based performance feedback vie short text messages and traditional postal letters on prescribing parenteral steroids by general practitioners- A randomized controlled trial. International Journal of Medical Informatics 2016; 87:36-43. <https://doi.org/10.1016/j.ijmedinf.2015.12.008>

**Pagaiya N, Garner P**. Primary care nurses using guidelines in Thailand: a randomized controlled trial. Tropical Medicine and International Helath 2005;10(5):471-477.

**Phuong HL, Nga TTT, Giao PT, Hung LQ, Bihn TQ, Nam NV, et al.** Randomised primary health center based interventions to improve the diagnosis and treatment of undifferentiated fever and dengue in Vietnam. BMC Health Services Research 2010;10:275. <https://doi.org/10.1186/1472-6963-10-275>

**Pinto D, Heleno B, Rodrigues DS, Papoila AL, Santos I, Caetano PA**. Effectiveness of educational outreach visits compared with usual guideline dissemination to improve family physician prescribing - an 18-month open cluster-randomized trial. Implementation Science 2018; 13:120. <https://doi.org/10.1186/s13012-018-0810-1>

**Ray WA, Stein CM, Byrd V, Shorr R, Pichert JW, Gideon P, et al**. Educational Program for Physiciand to Reduce Use of Noon-Steroidal Anti-Inflammatory Drugs Among Community-Dwelling Elderly Persons. A Randomized Controllod Trial. Madical Care 2000;39(5):425-435.

**Ruangkanchanasetr S**. Laboratory Investigation Utilization in Pediatric Out-Patient Department Ramathibodi Hospital. Journal of the Medical Association of Thailand = Chotmaihet thangpjaet 1993, 76, Suppl 2:194-208

**Sedrak MS, Myers JS, Small DS, Nachamkin I, Ziemba HB, Murray D,** et al. Effect of a Price Transparency Intervention in the Electronic Health Record on Clinician Ordering of Inpatient Laboratory Tests. The PRICE Randomized Clinical Trial. JAMA Intern Med. 2017;177(7):939-9445. doi:10.1001/jamainternmed.2017.1144

**Shojania KG, Yokoe D, Platt R, Fiskio J, Ma’luf N, Bates DW**. Reducing Vancomycin Use Utilizing a Computer Guideline: Results of a Randomized Controlled Trial. J AM Med Inform Assoc. 1998;5:554-562

**Smith TDH, Watt H, GunnL, Car J, Boyle RJ**. Recommending oral probiotics to reduce winter antibiotic prescriptions in people with astma: a pragmatic randomized controlled trial. Annals of Family medicine 2016;14:422-30 DOI: [10.1370/afm.1970](https://doi.org/10.1370/afm.1970)

**Solomon DH, van Houten L, Glynn RJ, Baden L, Curtis K, Schrager H, et al**. Academic Detailing to Improve Use of Broad-Spectrum Antibiotics at an Academic Medical Center. Arch intern Med. 2001;161:1897-1902.

**Soumerai SB, Salem-Schatz S, Avorn J, Casteris CS, Ross-Degnan D, Popovsky MA**. A Controlled Trial of Educational Outreach to Improve Blood Transfusion Practice. JAMA 1993;270:961-966

**Tan WJ, Acharyya S, Chew MH, Foo FH, Chan WH, Wong WK, et al**. Randomized control trial comparing an Alvarado Score-based management algorithm and current best practice in the evaluation of suspected appendicitis. World Journal of Emergency Surgery. 2020; 15:30. <https://doi.org/10.1186/s13017-020-00309-0>

**Tang Y, Liu C, Zhang X**. Public reporting as a prescriptions quality improvement measure in primary care settings in China: variations in effects associated with diagnoses. Sci Rep. 2016;6:39361 <https://doi.org/10.1038/srep39361>

**Tierney WM, McDonald CJ, Hui SL, Martin DK**. Computer Predictions of Abnormal Test Results. Effects on Outpatient Testing. JAMA 1988;259:1194-1198

**Tierney WM, Miller ME, McDonald CJ.** The effect on test ordering of informing Physicians of the charges for outpatient diagnostic tests. N Engl J Med. 1990;322:1499-504.

**Torrente F, Bustin J, Triskier F, Ajzenman N, Tomio A, Mastai R, et al**. Effect of a Social Norm Email Feedback Program on the Unnecessary Prescription of Nimodipine in Ambulatory Care of Older Adults. A Randomized Clinical Trial. JAMA Network Open 2020;3(12):e2027082. DOI: [10.1001/jamanetworkopen.2020.27082](https://doi.org/10.1001/jamanetworkopen.2020.27082)

**Wei X, Zhang Z, Hicks JP, Walley JD, King R, Newell JN, et al.** Long-term outcomes of an educational intervention to reduce antibiotic prescribing for childhood upper respiratory tract infections in rural China: Follow-up of a cluster-randomized controlled trial. PLos Med 2019;16(2):e1002733. https://doi.org/10.1371/journal. pmed.1002733

**Yang L, Liu C, Wnag L, Yin X, Zhang X**. Public reporting improves antibiotic prescribing for upper respiratory tract infections in primary care: a matched -pair cluster-randomized trial in China. Health Research Policy and Systems 2014;12:61. <https://doi.org/10.1186/1478-4505-12-61>

**Yip W, Powell-Jackson T, Chen W, Hu M, Fe E, Hu M, et al**. Capitation Combined Eith Pay-For-Performance Improves Antibiotic Prescribing Practices in Rural China. Health Aff 2014;33(3):502-10. DOI: [10.1377/hlthaff.2013.0702](https://doi.org/10.1377/hlthaff.2013.0702)

**Zwar N, Wolk J, Gordon J, Sanson-Fisher R, Kehoe L**. Influencing antibiotic prescribing in general practice: a trial of prescriber feedback and management guidelines. Family Practice 1999;16:495-500.
